# Successful childhood obesity management in primary care in Canada: what are the odds?

**DOI:** 10.7717/peerj.1327

**Published:** 2015-10-13

**Authors:** Stefan Kuhle, Rachel Doucette, Helena Piccinini-Vallis, Sara F.L. Kirk

**Affiliations:** 1 Perinatal Epidemiology Research Unit, Departments of Obstetrics & Gynaecology and Pediatrics, Dalhousie University, Halifax, Nova Scotia, Canada; 2 Department of Family Medicine, Dalhousie University, Halifax, Nova Scotia, Canada; 3 School of Health and Human Performance, Dalhousie University and the IWK Health Centre, Halifax, Nova Scotia, Canada

**Keywords:** Canada, Obesity, Primary care, Child, Barrier, Obesity management, Prevention, Health policy, Population health

## Abstract

**Background.** The management of a child presenting with obesity in a primary care setting can be viewed as a multi-step behavioral process with many perceived and actual barriers for families and primary care providers. In order to achieve the goal of behavior change and, ultimately, clinically meaningful weight management outcomes in a child who is considered obese, all steps in this process should ideally be completed. We sought to review the evidence for completing each step, and to estimate the population effect of secondary prevention of childhood obesity in Canada.

**Methods.** Data from the 2009/2010 Canadian Community Health Survey and from a review of the literature were used to estimate the probabilities for completion of each step. A flow chart based on these probabilities was used to determine the proportion of children with obesity that would undergo and achieve clinically meaningful weight management outcomes each year in Canada.

**Results.** We estimated that the probability of a child in Canada who presents with obesity achieving clinically meaningful weight management outcomes through secondary prevention in primary care is around 0.6% per year, with a range from 0.01% to 7.2% per year. The lack of accessible and effective weight management programs appeared to be the most important bottleneck in the process.

**Conclusions.** In order to make progress towards supporting effective pediatric obesity management, efforts should focus on population-based primary prevention and a systems approach to change our obesogenic society, alongside the allocation of resources toward weight management approaches that are comprehensively offered, equitably distributed and robustly evaluated.

## Introduction

Nearly one third of children in Canada is considered have overweight or obesity (Roberts et al., 2012). Obesity in childhood often tracks into adulthood (Singh et al., 2008) and increases the lifetime risk of cardiovascular, respiratory, orthopedic, gastrointestinal, and metabolic disease, among others (Thompson et al., 1999). Obesity is also shaped by a complex constellation of factors within the broader environment, which cannot by easily addressed through approaches aimed at individuals (Kopelman, Jebb & Butland, 2007). Examples include the ready availability of energy dense, nutrient-poor foods and increasingly sedentary lifestyles that make it challenging to adopt the behaviors required for weight loss to occur (August et al., 2008). Early identification, diagnosis, and management of childhood obesity (known as secondary prevention) are therefore important actions for reducing the burden of chronic disease and disability in adulthood (Lau et al., 2006). Primary care providers (PCP) play an important role in the diagnosis, education, and management of children who are obese as they commonly constitute the first point of contact within the health care system. A number of guidelines for the identification and management of children (and adults) with excess weight have been published over the last decade (August et al., 2008; Lau et al., 2006; Barlow, 2007; Canadian Task Force on Preventive Health Care, 2015). However, the management of excess weight in childhood is very complex, since the diagnosis may be associated with social stigma and challenging to discuss with the family (Oude Luttikhuis et al., 2009). Interventions are also more complex than a regular dose of medication, and the evidence for the effectiveness of interventions is still limited (Oude Luttikhuis et al., 2009).

**Figure 1:**
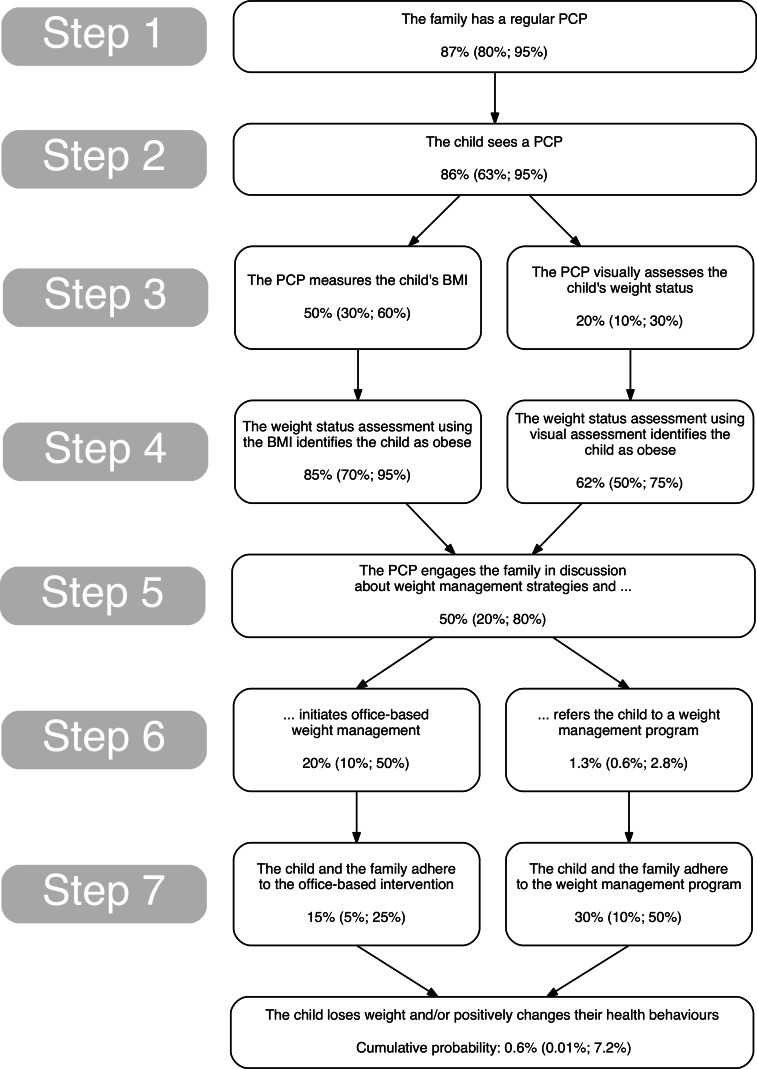
The steps involved in the assessment, diagnosis, and treatment of childhood obesity in primary care. Numbers indicate the estimated probabilities (and “worst case”/“best case” estimates) for completion of the respective step based on a literature review.

Based on existing best practice guidelines for pediatric obesity management (August et al., 2008; Lau et al., 2006; Barlow, 2007; Canadian Task Force on Preventive Health Care, 2015), the steps involved in the assessment, diagnosis, and treatment of obesity in children within primary care are typically conceptualized as follows (Fig. 1):

1. The family of a child with obesity has a regular PCP.
2. The child sees a PCP.
3. The PCP assesses the child’s weight status.
4. The weight status assessment identifies the child as obese.
5. The PCP engages the family in discussion about weight management strategies.
6. The PCP initiates office-based weight management or refers the family to a weight management program.
7. The child and the family adhere to the intervention and the intervention is effective in changing the child’s health behaviors and/or stabilizing/reducing the child’s weight.

In order for a child with obesity to fully benefit from weight management support, all steps in this process should be completed. This raises the question “what is the probability of achieving clinically meaningful weight management outcomes after completion of a weight management intervention in a primary care setting in Canada?”. We sought to review the evidence for the completion of each step of this process, and to estimate the population effect of secondary prevention of childhood obesity in Canada, so as to support decision-making regarding the successful management of pediatric obesity within primary health care.

## Materials & Methods

### Review of available evidence

Given the paucity of available datasets within clinical settings, we used the Public Use Microdata File of the Statistics Canada 2009/2010 Canadian Community Health Survey (CCHS) (Statistics Canada, 2011), a representative cross-sectional survey of Canadian residents aged 12 and older (*n* = 124,188). Analyses were performed in children and youth aged 12 to 17 years to estimate the proportion of children with obesity that have a PCP and the annual incidence of primary care visits (self-reported) in children who are obese (Steps 1 and 2).

For steps 3–7, we searched the literature using PubMed for English language articles on the assessment, diagnosis, initiation of intervention, and completion of weight management interventions in childhood published between January 2000 and April 2014. PubMed’s “Related citations” search was performed on relevant articles to identify additional studies. This process was repeated until no further relevant studies were identified. Studies were deemed relevant if they (1) were performed in Canada; (2) included participants between 5 and 18 years of age; (3) provided rates or proportions for the assessment, diagnosis, management, or treatment of childhood obesity in primary care. The full search strategy is available as a File S1.

### Determination of the population effect of secondary obesity prevention

Probability estimates for completion of each step were calculated as described above (Steps 1 and 2) or were extracted or estimated from the existing literature (Steps 3–7) by two of the authors (SK and SFLK). We converted annual rates of physician visits to probabilities using the standard formula *p* = 1 − *e*^(−rate × time)^ (Fleurence & Hollenbeak, 2007). In addition to the probability estimate for each step, we also used clinical judgment to choose two probabilities above and below the respective estimate so as to create a best and a worst case scenario. We calculated the cumulative probability of changing the child’s health behaviors and/or stabilizing/reducing the child’s weight by multiplying the probabilities at each stage within each of the four possible paths (see Fig. 1) and then calculating the sum of these four probabilities. This process was repeated using the worst and best case estimates. All analyses were performed in the statistical software package R (R Core Team).

## Results

### The steps in the weight management process

#### Step 1: the family has a regular primary care provider

The families of children with obesity who do not have a regular PCP will likely see PCPs only for acute care and therefore may never enter into the weight management process. According to the CCHS 2009/2010, 87% of children with obesity have a regular PCP. We used 95% and 80% as best and worst case estimates, respectively.

#### Step 2: the child sees a primary care provider

Children in the CCHS 2009/2010 with obesity reported on average 2 PCP visits over the past year. We used 3 and 1 visit(s) per year as best and worst case estimates.

#### Step 3: the primary care provider assesses the child’s weight status

Body mass index (BMI) calculation and comparison to age-related reference values is considered the recommended method to screen for excess weight in children, according to Canadian clinical practice guidelines (Lau et al., 2006; Canadian Task Force on Preventive Health Care, 2015). Self-reported data by Canadian PCPs shows that 60% of pediatricians and 30% of general practitioners use BMI based on measured height and weight to assess weight status in children and youth (He et al., 2010). A review of growth charts of children at five family physicians’ offices in Ontario found height and weight recorded in 59% of children between ages 2 and 6 years but only 39% had more than one BMI entry recorded (He & Sutton, 2004). Other methods to assess body weight include visual inspection, waist circumference, and weight-for-age (Dietitians of Canada, Canadian Paediatric Society, College of Family Physicians of Canada, Community Health Nurses Association of Canada, 2004; Spurrier, Magarey & Wong, 2006) but there are no estimates available on their frequency of use in Canada. Canadian guidelines for obesity management recommend measuring the waist circumference in adults if the BMI is >25 and ≤35 kg/m^2^ (Lau et al., 2006) but there is no such recommendation for children, possibly due to a lack of age-related reference values for waist circumference in children of all ages. We estimated that 50% of children would be likely to have their BMI measured at a PCP visit with 60% and 30% used as best and worst case estimates. We further estimated that 20% of children would have a visual assessment of their weight status (best case: 30%; worst case 10%).

#### Step 4: the weight status assessment identifies the child as obese

The current recommendation of the Canadian Pediatric Society is to use the WHO growth charts for BMI (De Onis et al., 2007) to identify obesity (Marchand & Members of the Collaborative Statement Advisory Group Nutrition and Gastroenterology Committee, 2010). However, BMI may not adequately account for the amount of visceral fat, which is more closely associated with cardiovascular disease markers and outcomes. The sensitivity of BMI (based on IOTF, WHO, or CDC obesity cutoffs) for identifying children with excess visceral fat reported in the literature ranges from approximately 70% to 95% (Harrington et al., 2013; Neovius et al., 2004; Mei et al., 2007; Freedman et al., 2005; Deurenberg-Yap et al., 2009). Based on the literature, we therefore used an 85% probability of correctly identifying a child with excess visceral fat mass, with 95% and 70% as the best and worst case estimates.

#### Step 5: the PCP engages the family in discussion about weight management strategies

After a diagnosis of obesity has been established, the PCP needs to raise the issue with the family to explore family attitudes towards and options for weight management. However, advice given during this discussion typically focuses on the provision of information (what to eat, how much to exercise, etc.) and does not constitute a formal weight management intervention. A recent Canadian survey of PCPs in Canada showed that 85% of physicians who responded to the survey routinely give parents of children with obesity and overweight advice about diet and physical activity during office visits (He et al., 2010). Since the authors excluded missing responses from the analysis and there is potential for social desirability bias and selection bias, this is likely an overestimate. Therefore, we used a probability of 50%, with 80% and 20% as the best and worst case estimates, for engaging the family in discussion about weight management.

#### Step 6: the primary care provider initiates office-based weight management or refers the family to a weight management program

Canadian clinical practice guidelines on the management and prevention of obesity recommend a lifestyle intervention that aims at improving diet and physical activity as the first line of management for pediatric obesity (Lau et al., 2006; Canadian Task Force on Preventive Health Care, 2015). An intervention can be administered by the PCP or through enrollment in a weight management program. There is no information available on how many PCPs in Canada have the necessary training for delivering the intervention or how often a structured, tested, and effective approach is used. We estimated that only 20% of PCP has the training, time, and resources to initiate a structured in-office lifestyle intervention (best case 50%, worst case 10%).

The options for referring a child with obesity to a weight management program are very limited. Within Canada, there are 23 childhood obesity management programs registered with the Canadian Obesity Network’s Weight Management Registry as of 2014 (Canadian Obesity Network). All programs use a multidisciplinary approach with a combination of behavioral lifestyle interventions, structured exercise plans, energy reduced diets, pharmacotherapy, or bariatric surgery. There is a lack of standardization among intervention components and program structure, and currently few programs have published evaluations (Ball, Ambler & Chanoine, 2011; Watson-Jarvis, Johnston & Clark, 2011; Panagiotopoulos et al., 2011). The 15/23 programs that reported enrollment figures treat between 10 and 3,000 patients annually with the mean and median number of patients per program being 304 and 68 (Canadian Obesity Network). The number of children between 5 and 17 years of age in Canada who were considered obese in 2013 was approximately 568,000 (Roberts et al., 2012; Statistics Canada). Of these, approximately 121,000 (20.6%) would complete the first five steps, resulting in a probability of 1.3% (median of 68 spots per year × 23 centres/121,000) for being referred (and admitted) to a weight management program. We used 2.8% (150 spots per year) and 0.6% (30 spots per year) as best and worst case estimates.

#### Step 7: the child and the family adhere to the intervention and the intervention is effective in changing the child’s health behaviors and/or reducing or stabilizing the child’s weight

Weight management interventions have traditionally been evaluated based on participants’ reductions in BMI after completion of the intervention. More recently, program evaluations have begun to focus on sustainable changes in health behaviors and markers of chronic disease as indicators of effectiveness. Thus, for the purposes of this study it is difficult to determine from the available literature what proportion of children actually achieve clinically meaningful weight management outcomes after completion of a weight management intervention.

Evidence on the effectiveness of office-based lifestyle counseling is limited given the often non-standardized approach. As there are no Canadian data on the effectiveness of office-based counseling for children or adults with obesity we used the “5-2-1-0” strategy developed in the US as a best practice model of a structured approach to office-based lifestyle counseling that has been applied within primary care and is described in the literature (Chang et al., 2010; Polacsek et al., 2009). This strategy encourages children to meet evidence-based behavioral targets daily within a family focused intervention: eat at least five servings of fruit or vegetables per day; limit screen time to less than two hours per day; participate in physical activities for at least one hour per day; consume zero or minimal sweetened drinks (Polacsek et al., 2009). Data from 12 PCP intervention sites in urban and rural areas of Maine demonstrated behavior changes between 12% and 26% based on parental report (Polacsek et al., 2009). Two studies from Minnesota that used the “5-2-1-0” behavioral modification program in primary care reported adherence numbers for the full program of 6/68 (9%) (Kwapiszewski & Lee Wallace, 2011) and 40/70 (57%) respectively (Tucker et al., 2013). In the former study, average BMI reduction was 0.43% of baseline BMI, while decreases in BMI were reported for 28% of the children in the latter study. A recent Cochrane review identified 17 interventions for childhood obesity in primary care, 12 of which reported a significant effect immediately following the intervention. Seven out of 17 studies maintained the effect for months to years post intervention (Sargent, Pilotto & Baur, 2011). Effect sizes ranged from small decreases in dietary sugar intake to substantial drops in overweight prevalence. Based on the above literature, we estimated that the probability of achieving clinically meaningful weight management outcomes as a result of a structured or unstructured lifestyle intervention in the PCP office is 15% with best and worst case estimates of 25% and 5%.

Four Canadian weight management programs have published evaluations, which demonstrated statistically significant, clinically moderate weight loss effects but high rates of attrition (Watson-Jarvis, Johnston & Clark, 2011; Panagiotopoulos et al., 2011; Ball et al., 2011; Avis et al., 2013). The Centre for Healthy Weights Shapedown BC obesity management program reports a significant change from weight gain to weight loss at the end of the program. Measured as monthly percentage weight change, youth in this program went from 0.89% weight gain at the start of the program to 0.37% weight loss at completion. There were also significant improvements in fasting insulin levels, physical activity levels and measures of mental wellbeing. However, only 32.8% of participants (39/119) attended all 10 weekly group sessions (Panagiotopoulos et al., 2011). A randomized controlled trial of two one-on-one lifestyle interventions in a weight management clinic in Edmonton, Alberta, showed modest short-term decreases in BMI-z scores of 3.9% and 6.5% compared to a wait list control group. Attrition rates in both intervention groups were around 40% over the 16–20 week program, and were highest shortly after the initiation of the program. However, those who completed the program had a high degree of participation (Ball et al., 2011). A family-focused, behavior-based education program in Calgary had 78% completion rate (271/345) over the course of 8–12 weeks. Participant BMI z-scores on average decreased from 2.14 to 2.08 (Watson-Jarvis, Johnston & Clark, 2011). Program attrition in an interdisciplinary, individualized care weight management program in Edmonton, Alberta, was 49 and 73% at 7 and 11 months, with 8 and 5% of children showing BMI decreases at these time-points (Avis et al., 2013). Given the considerable heterogeneity between studies with regard to sample characteristics, settings, interventions, and outcome measures, we estimated the probability of weight loss after completion of a lifestyle intervention at 30% with 10% and 50% as worst and best case estimates.

### Cumulative probability of successful childhood obesity management

The estimated probabilities from the literature for the completion of each step of the weight management process are shown in Fig. 1. We estimated that the probability of a child in Canada with obesity achieving a healthy weight or improved health behaviors through secondary prevention in primary care to be around 0.6% per year, with a range from 0.01% to 7.2% per year. By way of example, out of 1,000 Canadian children, on average 117 will have obesity (Roberts et al., 2012), and of these, one child (worst case: none, best case: eight children) is estimated to achieve a healthy weight or improved health behaviors each year through primary care-initiated weight management intervention.

## Discussion

In this present study, we have examined the process of weight management in primary care and have proposed a model to describe this process. Based on estimates derived from a review of the literature, the probability of positively changing health behaviors with obesity and/or achieving clinically meaningful weight loss is currently very low. Our study has identified multiple targets for improvement of weight management outcomes but there are some limitations that should be acknowledged. Most importantly, the estimates for successful weight management presented in this study, while based on evidence available from the literature, may not be accurate as the probabilities for completion of each step were difficult to estimate due to heterogeneity of study populations, interventions, and outcome measures that are currently presented in the literature. Our calculation of the net population effect of secondary obesity prevention does not take into account factors that may modify the chance of success, such as actual BMI, existence of comorbidities, age, gender, parental weight status, area of residence, or access to care. Considering these factors would require a complex microsimulation model, incorporating more detailed data than is currently available and therefore relying on more assumptions. Whether this would change the conclusion reached in our approach—that childhood obesity management under the current primary care model will only help a very small number of children—is therefore not known. The underlying assumption of our calculations—that the probabilities for completion of each step are independent from each other—is likely not the case in actuality. However, this analysis has further highlighted the complexity of childhood weight management, the potential for failure at multiple points in the process, and the potential for substantial impact on outcomes should all the steps outlined in theory actually be completed in practice.

Relational continuity of care is critical for the effective and successful management of childhood obesity in primary care as it allows PCPs to integrate the proximal and distal contexts of the child and family into the management plan. For 13% of Canadian families, who access primary care through walk-in clinics, emergency rooms, or who may regularly change GPs, the lack of continuity of care provides the first major barrier for the management of childhood obesity (and other health conditions, for that matter). While the data from the CCHS do not provide information on the reason for attending a PCPs office, visits that are primarily motivated by concerns about the child’s weight are likely rare. Data from Canada (Chaimovitz et al., 2008) and other countries (Jeffery et al., 2005; West et al., 2008; Wake, 2009) show that parents are often incorrect in their perception of their child’s weight status and tend to underestimate their child’s weight, especially if they have overweight or obesity themselves (Chaimovitz et al., 2008; Jeffery et al., 2005). Jeffery et al. (2005) suggested that the lack of awareness may be due to denial or a desensitization to excess weight given the high prevalence of obesity. Parents are “critical partners” (Jeffery et al., 2005) for PCPs in the management of childhood obesity, and their lack of awareness or concern with their child’s weight status constitutes a major barrier to identification of obesity as well as to initiating and sustaining a change in lifestyle behaviors.

Calculation of BMI based on measured height and weight in children and adults during PCP visits has been recommended by various medical professional bodies and task forces in Canada (Lau et al., 2006; Canadian Task Force on Preventive Health Care, 2015; Dietitians of Canada, Canadian Paediatric Society, College of Family Physicians of Canada, Community Health Nurses Association of Canada, 2004; Fitzpatrick et al.) but the implementation in clinical practice has been limited. Of the children who do have a regular PCP, 60% or fewer will have their weight status measured or recorded during a visit (He et al., 2010; He & Sutton, 2004). In primary care practices where BMI is not regularly recorded, physicians named a lack of familiarity with BMI, lack of agreement with the use of BMI as a screening tool, limited time during appointment, and skepticism about treatment effectiveness as barriers (Flower et al., 2007). There is some evidence that rates of BMI measurement may improve through provider education, clinical practice tools, and the use of electronic medical records that prompt for regular weight measurements or automatically calculate the BMI percentile (Piccinini-Vallis, 2011; Dunlop et al., 2007; Savinon et al., 2012; Keehbauch et al., 2012; Bode, Roberts & Johnson, 2013).

The most important barrier to managing pediatric obesity in primary care, as identified by more than 70% of respondents in the survey of survey of Canadian PCPs by He et al. (2010), was the ‘obesogenic environment’. An obesogenic environment is typically defined as ‘the sum of influences that the surroundings, opportunities, or conditions of life have on promoting obesity in individuals or populations’ (Swinburn, Egger & Raza, 1999). Other barriers included time constraints, lack of training, support, and options for referral, parents with obesity who are perceived to be poor role models, lack of patient motivation to change behaviors, and poor compliance with recommendations for change. Physicians were also concerned that they may interfere with family function, contribute to stereotypes, negatively influence a patient’s self esteem, or precipitate an eating disorder in a child with obesity (He et al., 2010). These barriers and concerns are echoed in studies from other countries (Story et al., 2002; Gage et al., 2012; Dettori et al., 2009), highlighting a pressing need for a more holistic approach to obesity management and prevention, that recognizes the complex constellation of factors in its and shifts the focus from health not weight as an outcome.

We believe that engaging the family in discussion about the child’s weight and initiating office-based counseling are distinct issues and hence we separated the two in our process model. While the majority (85%) of PCPs in the survey by He et al. (2010) stated that they routinely give parents of children with obesity advice about diet and physical activity during office visits, this likely does not constitute a formal weight management intervention. Our literature search did not identify any studies that provided information on how often a formal office-based behavior modification intervention is initiated by PCPs in Canada. If we use documentation of a diagnosis of obesity, either on the chart or on the billing form, as a proxy indicator for discussing the issue with the family, the actual proportion of children with obesity that receive an office-based weight management intervention is likely very low. A Canadian study linking a population-based survey with physician billing data showed that only 10% of children aged 10–11 years with a BMI, based on measured height and weight, that identified them as having obesity received an ICD code diagnosis of obesity during the same year, with a quarter of children with obesity that did not have an obesity diagnosis having a BMI between 28.5 and 44.0 kg/m^2^ (Kuhle et al., 2011). According to US studies, documentation of a diagnosis of obesity on the charts ranged from 18 to 66% of children who were identified with obesity based on their measured BMI (O’Brien, Holubkov & Reis, 2004; Dorsey et al., 2010; Dilley et al., 2007; Patel et al., 2010). There is also a need to consider whether an additional step be included, given that there is increasing evidence that parents may not accurately perceive the weight status of their child(ren). For example, a recent large meta-analysis (Lundahl, Kidwell & Nelson, 2014) revealed that half the parents included in the studies reviewed underestimated their children’s overweight/obesity status and a significant minority underestimated children’s normal weight. This has important implications because a substantial number of children might not even have the opportunity to enter into the weight management process outlined.

One of the main barriers reported by the majority of respondents in the survey of PCPs in Canada was the lack of options for referral (He et al., 2010). The programs registered with the Canadian Obesity Network are distributed in major cities and are mostly associated with hospitals and academic centers, which provides a barrier to access for people living outside the urban core. For example, one in five patients referred to a weight management program in Edmonton, AB, lives more than a one-hour drive away from the program location (Ambler, Hagedorn & Ball, 2010). There are programs in most Canadian provinces, with the exception of Saskatchewan, Nova Scotia, and Prince Edward Island, and there are currently no programs in any of the three territories, highlighting that programs are lacking in regions where they are likely needed the most (Shields & Tjepkema, 2006; Statistics Canada: Canadian Community Health Survey: a first look). The limited access to weight management programs in Canada puts the onus on PCPs to initiate lifestyle interventions in the office. It also highlights the need for population health interventions that have a greater chance of impact on the behaviors (i.e., diet and physical activity interventions) that influence health across whole populations rather than focusing on obesity only.

There are limitations to our approach that should be considered. First, we did not consider pharmacotherapy or bariatric surgery as intervention options in our model as they are only indicated in a small proportion of children with obesity and their long-term success is dependent on concurrent support by an interdisciplinary weight management program (Davies et al., 2014). The outcomes of the weight management program evaluations reported in the literature vary considerably and include health behaviors, cardiovascular disease markers, and various measures of body fatness, which hampered any meaningful estimation of what could be considered to be a successful outcome. Moreover, we found no consistent definition of what success in pediatric weight management actually means or any studies that considered weight maintenance as an outcome. Moreover, the follow-up period of the studies was often limited to a few weeks or months and since there are no data available on the sustainability of the outcomes of Step 7, the long-term success rates may even be lower than indicated. Although the steps upon which we based our estimates are derived from existing guidelines and recommendations, they are open to criticism, since there are likely many other approaches to management of obesity in childhood; this is a fast moving field where evidence is emerging constantly. There remain a number of challenges with defining successful weight management for pediatric obesity, and a lack of data to inform this debate. We do not seek to provide the “only approach”, but to illustrate the flaws within the steps that are typically considered on the management pathway for childhood obesity, particularly in Canada, and upon which existing management guidelines are typically based. Other approaches are in existence, e.g., the 4-step approach outlined by Spear et al. (2007). Ours is just one approach that we hope will stimulate discussion regarding the capacity of the primary (and other components of the) health care system to address pediatric obesity, and to what extent each of the steps listed might be operationalized. We also acknowledge that other countries and jurisdictions may have different experiences that could alter these conclusions, although there is evidence from European studies that suggest similar barriers are encountered within primary care (Van Gerwen et al., 2009; Mazur et al., 2013).

## Conclusions

Within the prevailing model of care, and as outlined in the model described in this paper, we predict that only a very small fraction of children with obesity will achieve a healthy weight through a primary care weight management intervention in Canada. The lack of accessible and effective weight management programs appears to be the most important bottleneck in the process. While the optimal process to manage childhood obesity in primary care remains open to debate, our findings point to the need for greater effort to be focused on population-based primary prevention and a systems approach to change our obesogenic society. There is also a need to allocate additional resources toward evidence-based obesity and outcomes that are not primarily weight-focused management initiatives for children that are comprehensively offered, equitably distributed and robustly evaluated.

## Supplemental Information

10.7717/peerj.1327/supp-1 File S1 Literature search strategy

## Additional Information and Declarations

### Data Availability

The following information was supplied regarding data availability: All the data that were used in the analysis can be found in the Results section and in Fig. 1.
