# Supplementary material for: Successful childhood obesity management in primary care in Canada: what are the odds?
Source: PeerJ. 2015 Oct 13;3:e1327. doi: 10.7717/peerj.1327 (PMC4662587; doi:10.7717/peerj.1327)
Supplement: File S1 [file peerj-03-1327-s001.docx]

**Literature search strategy**

The following text words plus relevant subject headings and a search filter for Canadian studies were used. Applicable variations of the words and phases were also used.

**Step 3**

(child OR preschooler OR infant OR adolescent OR pediatric OR youth)

AND (primary health care OR family physician OR general practitioner OR pediatrician OR family doctor OR primary care)

AND (visit OR appointment OR well child OR child health)

AND (body mass index OR BMI OR weight OR height OR growth OR anthropometry OR quetelet)

AND (measurement OR assessment OR screening OR physical exam OR growth charts OR physician practice patterns)

**Steps 4 and 5**

(child OR preschooler OR infant OR adolescent OR pediatric OR youth)

AND (primary health care OR family physician OR general practitioner OR pediatrician OR family doctor OR primary care)

AND (documented OR diagnosis OR identification OR medical record OR physician practice patterns)

AND (overweight OR obesity)

**Step 6**

(child OR preschooler OR infant OR adolescent OR pediatric OR youth)

AND (primary health care OR family physician OR general practitioner OR pediatrician OR family doctor OR primary care)

AND (overweight OR obesity)

AND (intervention OR obesity therapy OR overweight therapy OR weight management OR weight reduction program OR weight loss OR counseling OR diet therapy OR drug therapy OR behavior therapy OR behavior modification OR cognitive therapy OR lifestyle modification OR nutrition OR Canada Food Guide OR diet OR health promotion OR exercise OR physical activity OR family therapy OR group therapy)

**Step 7**

(child OR preschooler OR infant OR adolescent OR pediatric OR youth)

AND (overweight OR obesity)

AND (intervention OR obesity therapy OR overweight therapy OR weight management OR weight reduction program OR weight loss OR counseling OR diet therapy OR drug therapy OR behavior therapy OR behavior modification OR cognitive therapy OR lifestyle modification OR nutrition OR Canada Food Guide OR diet OR health promotion OR exercise OR physical activity OR family therapy OR group therapy)

AND (adherence OR patient compliance OR completion)
